# Curcumin Mitigates the High-Fat High-Sugar Diet-Induced Impairment of Spatial Memory, Hepatic Metabolism, and the Alteration of the Gut Microbiome in Alzheimer’s Disease-Induced (3xTg-AD) Mice

**DOI:** 10.3390/nu16020240

**Published:** 2024-01-12

**Authors:** Gopal Lamichhane, Jing Liu, Su-Jeong Lee, Da-Yeon Lee, Guolong Zhang, Yoo Kim

**Affiliations:** 1 Department of Nutritional Sciences, Oklahoma State University, Stillwater, OK 74078, USA; gopal.lamichhane@okstate.edu (G.L.); crystal.lee10@okstate.edu (S.-J.L.); dayeon.lee@okstate.edu (D.-Y.L.); 2 Department of Animal and Food Sciences, Oklahoma State University, Stillwater, OK 74078, USA; jing.liu12@okstate.edu (J.L.); glenn.zhang@okstate.edu (G.Z.)

**Keywords:** curcumin, high-fat high-sugar diet, Alzheimer’s disease, metabolic disease, 3xTg-AD mice, gut microbiota, brain–gut axis, liver–gut axis

## Abstract

The escalating prevalence of metabolic diseases and an aging demographic has been correlated with a concerning rise in Alzheimer’s disease (AD) incidence. This study aimed to access the protective effects of curcumin, a bioactive flavonoid from turmeric, on spatial memory, metabolic functions, and the regulation of the gut microbiome in AD-induced (3xTg-AD) mice fed with either a normal chow diet (NCD) or a high-fat high-sugar diet (HFHSD). Our findings revealed an augmented susceptibility of the HFHSD-fed 3xTg-AD mice for weight gain and memory impairment, while curcumin supplementation demonstrated a protective effect against these changes. This was evidenced by significantly reduced body weight gain and improved behavioral and cognitive function in the curcumin-treated group. These improvements were substantiated by diminished fatty acid synthesis, altered cholesterol metabolism, and suppressed adipogenesis-related pathways in the liver, along with modified synaptic plasticity-related pathways in the brain. Moreover, curcumin enriched beneficial gut microbiota, including *Oscillospiraceae* and *Rikenellaceae* at the family level, and *Oscillibacter*, *Alistipes*, *Pseudoflavonifractor*, *Duncaniella*, and *Flintibacter* at the genus level. The observed alteration in these gut microbiota profiles suggests a potential crosswalk in the liver and brain for regulating metabolic and cognitive functions, particularly in the context of obesity-associated cognitive disfunction, notably AD.

## 1. Introduction

The global burden of metabolic diseases has surged over the past two decades, with projections indicating a continuous rise without proper intervention [1]. Lifestyle factors such as an improper diet and sedentary habits contribute to metabolic disorders, encompassing cardiovascular problems, insulin resistance, hyperglycemia, impaired glucose tolerance, adiposity, and high blood pressure. Importantly, these factors are interconnected and are considered risk factors for Alzheimer’s disease (AD) [2]. The Western diet, characterized by high saturated fat and a high sugar content, has been implicated in promoting AD-related neuropathology. This diet is associated with reduced neurogenesis, increased hippocampal inflammation, and enhanced blood–brain barrier permeability, leading to the accumulation of toxic amyloid beta (Aβ) peptides and other substances affecting cognition [3,4,5]. Several epidemiological data also support this, as research has shown a reduced hippocampal volume [6], an increased AD risk [7,8] and a high odds ratio for developing tubulin-associated unit (tau) pathology and preclinical AD with the consumption of a Western diet [9]. Conversely, Mediterranean and other healthy dietary patterns have been found to offer protection [8,10]. Additionally, nonalcoholic fatty liver disease (NAFLD), a common consequence of obesity resulting from a high-fat high-sugar diet (HFHSD), increases the risk of dementia by altering the brain–blood flow and is predisposed to stroke, asymptomatic brain injury, brain aging, and cognitive decline [11]. The liver also plays a crucial role in the peripheral clearance of Aβ, influencing its increased efflux from the brain and reducing the Aβ load [12,13]. However, NAFLD disrupts this process, resulting in increased Aβ- and tau loads, as well as neuroinflammation [14]. Furthermore, disruptions in insulin signaling transduction and mitochondrial dysfunction have been observed in the hippocampus of AD mice [15]. Proper dietary changes, environmental control, and increased physical activity have shown promise in ameliorating these metabolic abnormalities and AD symptoms [16], emphasizing the need for a common approach to address these issues.

With the advancements in medical treatment and care, the life expectancy of humans has increased, leading to a growing elderly population with a higher risk of developing metabolic diseases and dementia [17,18]. AD, the most common form of dementia, is a progressive neurodegenerative disorder marked by behavioral alterations and cognitive decline [19]. Pathologically, AD is characterized by the accumulation of neuritic plaques and neurofibrillary tangles composed of Aβ peptides and tau proteins, respectively [19]. Environmental factors, such as an improper diet and a lack of exercise, can induce epigenetic changes in the AD-associated pathways, predisposing individuals to AD [20]. AD accounts for approximately 60–70% of dementia cases, ranking as the seventh leading cause of death and a major contributor to dependency and disabilities among the elderly worldwide [21]. The prevalence of AD-associated dementia is projected to increase significantly, reaching 13.8 million individuals aged 65 or older in the United States by 2060 [22].

Growing evidence has highlighted the pivotal role of the gut microbiome in AD pathology. Specific changes in gut microbiota have been identified in both preclinical and clinical AD, suggesting that the microbiome profile can serve as a marker of preclinical AD [23]. Microbiome-derived metabolites can influence peripheral immune cell functions and cytokine levels, modifying the blood–brain barrier and neuroinflammatory response. Hormonal and neuroactive metabolites produced by the gut microbiome can directly impact brain functions through the vagus nerve. The modulation of amyloidosis by altering the gut microbiome composition and diversity has been observed in germ-free mouse models and fecal matter transplantation [24]. These findings support the existence of the gut–brain axis, suggesting that modulating the gut microbiome diversity and abundance may provide a promising avenue for effective interventions in AD.

Curcumin, a major bioactive chemical constituent derived from turmeric, has been found to exhibit a range of neuroprotective effects, including the reduction in the amyloid burden, neuroinflammation, oxidative stress, infection, and inflammation [25]. Previous findings also provided evidence of the modulatory role of curcumin in mediating several targets of metabolic diseases [26]. Reduced plasma glucose and triglyceride [27] and improved β cell function and afamin levels were also evident after curcumin consumption in some clinical trials [28]. Our previous studies have demonstrated the protective effects of curcumin on metabolic dysfunctions such as body weight gain, liver fat accumulation, and dysregulated insulin homeostasis in HFHSD-fed middle-aged and old mice [29,30,31]. However, there was no clear understanding on how curcumin ameliorates the metabolic abnormality-induced AD-related pathologies in HFHSD-fed 3xTg-AD mice. This gap in knowledge motivated our investigation to find whether the increased vulnerability of AD symptoms due to HFHSD can be mitigated with curcumin supplementation. In this study, we also aimed to assess the changes in the gut microbiome after curcumin consumption and its implications for metabolic diseases and AD, with the goal of establishing any cross-interaction of the gut microbiome with the liver and brain.

## 2. Materials and Methods

### 2.1. Animals and Treatment

Seven-month-old young female 3xTg-AD mice and the control (B6129SF2/J) mice were purchased from Jackson Laboratory (Bar Harbor, ME, USA) and acclimatized for a week with standard chow diet feeding. The baseline parameters, including the body weight and fed and fasting blood glucose levels, were measured. Both the control and transgenic mice were randomly assigned to four groups: normal chow diet (NCD), curcumin-supplemented (4 g/kg) normal chow diet (NCD + CUR), high-fat high-sugar diet (HFHSD), and curcumin-supplemented (4 g/kg) high-fat high-sugar diet (HFHSD + CUR)-fed groups. Dietary intervention was conducted for 14 weeks. Customized NCDs and HFHSDs with and without curcumin (Sigma-Aldrich, St. Louis, MO, USA) were ordered from Dyets Inc. (Bethlehem, PA, USA). The mice were provided *ad libitum* access to food and water throughout the study period, and the body weight and food intake were monitored weekly.

### 2.2. Glucose and Insulin Tolerance Test

The mice were fasted for a 16-h period before the glucose tolerance test (GTT) at week 13. A total of 2 g/kg of a glucose solution (Alpha Teknova Inc., Hollister, CA, USA) was administrated via intraperitoneal injection, and the blood glucose levels were measured at 0 min and 15, 30, 60, 90, and 120 min post-injection using the Contour ^®^ next EZ glucometer (Parsippany, NJ, USA).

For the insulin tolerance test (ITT) at week 14, the mice were fasted for 6 h before receiving intraperitoneal insulin (Novolin R from Novo Nordisk, Bagsvaerd, Denmark) at dose of 0.75 IU/kg. The blood glucose levels were measured at 0 min and 15, 30, 60, 90, and 120 min post-injection.

### 2.3. Behavioral Test

#### 2.3.1. Y-Maze Test

Spatial recognition memory was assessed using the Y-maze test at week 11 following the methods explained previously [32]. The mice were acclimatized to the test room for 1 h before training. One arm of Y-maze was closed, and each mouse was placed at the end of one open arm, facing the center. Their movements were recorded during a 15-min training period, and the mice was subsequently tested for 5 min with the previously closed arm now open. Their movements were tracked using a camera connected to a laptop using the ANY-maze software (v 7.2, 64 bits, Stoelting Co., Wood Dale, IL, USA) to evaluate the entries, time spent, and alterations.

#### 2.3.2. Open Field Test (OFT)

The open field test was conducted on the 10th week to evaluate the behavioral and locomotive alterations [33]. The mice were acclimatized to the test environment for 1 h, and their movements were monitored for 5 min using a camera connected to a computer using the ANY-maze software (v 7.2). The number of entries to the central and corner zones, the time spent in each zone, latency, and the total travel distance were recorded.

#### 2.3.3. Novel Object Recognition (NOR) Test

The novel object recognition test, conducted as previously described [34], involved habituating the mice to a rectangular apparatus for 5 min, followed by an exploration of the arena with two identical objects during a 5-min training session. After 3 h, the mice were reintroduced to the arena with one identical and one new object. The object exploration was recorded using a digital camera connected to a computer using the ANY-maze software (v 7.2).

### 2.4. Gene Profiling Analysis in the Liver and Hippocampus

The liver and hippocampus samples were preserved in RNAlater^TM^ (R0901, Millipore Sigma, St. Louis, MO, USA), and RNA isolation, cDNA library preparation, and RNA sequencing were performed by Novogene Co Ltd. (Sacramento, CA, USA). The sequencing data were deposited into the NCBI Sequence Read Archive database under the BioProject accession number PRJNA1052364. Prior to assembly, the adapters were trimmed, and the quality was assessed for raw reads using Trimmomatic (v0.39). The reads with low-quality base calls (phred score ≤ 20) were removed using SolexaQA++ (v.3.1.7). The preprocessed fastq files were then aligned to the GRCm39 reference genome (C57BL/6J strain) using Hisat2 (v2.2.1) with default parameters and the SAM files were transformed into a sorted BAM format using SAMtools (v1.6.0). The transcriptional coordinates and expression values were generated as individual GTF files using StringTie (v2.1.4).

The transcript expression files were exported as read count matrices from the GTF files and a differential expression analysis was performed by edgeR (v3.32.1) after the filterByExpr function was used to remove the low-expressed genes. The genes with a *p*-value < 0.01, log2 fold changes > 1 were considered as DEGs. The Gene Kyoto Encyclopedia of Genes and Genomes (KEGG) pathways network and gene set enrichment (GSE) were identified using Goseq and clusterProfiler packages. A *p*-value < 0.05 was considered as significant.

### 2.5. Microbial Analysis of the Feces

The feces samples were collected and stored at −80 degree Celsius until genomic DNA (gDNA) isolation using the QIAamp Fast DNA Stool Mini Kit from Qiagen (Hilden, Germany). The isolated gDNA was then sent to Novogene for 16S rRNA sequencing. The sequencing data were deposited in the NCBI Sequence Read Archive database under the same BioProject accession number as the RNA sequencing data (PRJNA1052364). The raw sequencing reads were analyzed using the QIIME 2 pipeline (v. 2020.11). Briefly, the adaptor and primer sequences were removed from each read using the cut-adapt plugin. The paired-end reads were then merged using ‘vsearch join-pairs’ and the low-quality reads were filtered out using a ‘quality filter q-score’. The sequences were trimmed to 402 nucleotides and denoised using Deblur. The resulting sequences were then classified into bacterial ASVs (amplicon sequence variants) using the RDP 16S rRNA training set (v. 18) and Bayesian classifier. A bootstrap confidence of 80% was used for taxonomic classification. The ASVs with a classification of <80% were assigned the name of the last confidently assigned level followed by “unidentified”. The ASVs appearing in <5% of the samples were removed from the analysis. The top 20 ASVs and all the differentially enriched bacteria were further confirmed and reclassified, if necessary, based on a more recent EzBioCloud 16S database (v. 2021.07.07). The data were normalized using cumulative sum scaling (CSS) in the metagenomeSeq package of R (v. 1.4.0).

The α-diversity (Shannon’s Index, Observed ASVs, and Pielou’s Evenness) and β-diversity (unweighted and weighted UniFrac distances) were calculated using the phyloseq package in R (v. 1.42.0). The statistical significance in α-diversity and relative abundance were determined using a nonparametric Kruskal–Wallis test. The significance in the β-diversity was determined using a nonparametric permutational multivariate analysis of variance (PERMANOVA) with the adonis function in the vegan package (v. 2.6.4) [16]. The differential enrichment of the bacterial features was determined using the linear discriminant analysis (LDA) effect size (LEfSe), with the all-against-all multiclass analysis, *p* < 0.05, and a logarithmic LDA threshold of 3.0.

### 2.6. Real Time Quantitative Polymerase Chain Reaction (qPCR)

The total RNA was isolated using the TRIzol Reagent (Thermo Fisher Scientific, CA, USA), and cDNA was synthesized using the iScript™ cDNA Synthesis Kit (Bio-Rad Laboratories, Inc., Hercules, CA, USA). The expression of the gene was assessed using the SYBR^®^ Green PCR Master Mix (Applied Biosystems; Thermo Fisher Scientific, Waltham, MA, USA) on a CFX Opus 384 Real-Time PCR System (Bio-Rad Laboratories). The data were normalized to 18S ribosomal RNA and β-actin.

### 2.7. Statistical Analysis

All the data were analyzed using GraphPad Prism (V 9.5.1: GraphPad Inc., Boston, MA, USA). A two-way ANOVA followed by Tukey’s multiple comparison tests were used for the ITT and GTT. A one-way ANOVA was used for body weight gain and alteration percentage. An unpaired *t*-test was used for analyzing the areas under the curve (AUCs) of the ITT and GTT, the novel entries in Y-maze, and the qPCR results. The data were presented as the mean ± SEM, and a statistical significance was indicated as * *p* < 0.05, ** *p* < 0.01, and *** *p* < 0.001.

## 3. Results

### 3.1. Curcumin Attenuated HFHSD-Induced Body Weight Gain in 3xTg-AD Mice

**Figure 1:**
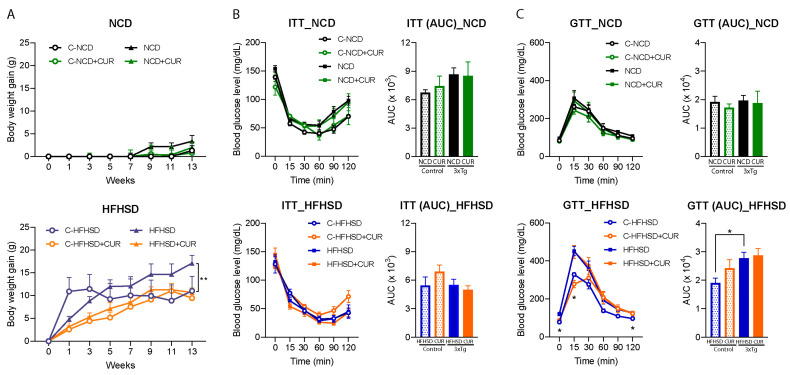
Effects of curcumin supplementation on body weight gain, the ITT, and the GTT in the control and 3xTg-AD mice. (**A**) Curcumin reduced body weight gain in the HFHSD-fed 3xTg mice (*n* = 5 to 6; one-way ANOVA followed by Tukey’s multiple comparison test; ** *p* < 0.001) in a 14-week intervention period compared to the HFHSD-fed 3xTg mice; (**B**) the ITT and AUC reflected the effect of curcumin on the insulin sensitivity of the NCD-fed mice (up) and HFHSD-fed mice (down) (*n* = 4 to 6, two-way ANOVA followed by Tukey’s multiple comparison test); (**C**) the GTT and AUC reflected the effect of curcumin on the glucose disposal of the NCD-fed mice (up) and HFHSD-fed mice (down) (*n* = 4 to 6, two-way ANOVA followed by Tukey’s multiple comparison test * *p* < 0.05 vs. the HFHSD control; the AUC between the groups were compared using an unpaired *t*-test; * *p* < 0.05). The groups are represented as C-NCD: NCD-fed control mice; C-HFHSD: HFHSD-fed control mice; NCD: NCD-fed 3xTg mice, and HFHSD: HFHSD-fed 3xTg-AD group. The mice that did not show positive weight gain from HFHSD feeding were excluded from the study.

This study investigated the impact of dietary interventions with and without curcumin supplementation on the body weight gain of both a control and 3xTg-AD female mice over a 14-week period (Figure 1A). The HFHSD + CUR-fed 3xTg mice showed a significantly lower average body weight gain (10.65 ± 0.41 g; *p* < 0.01) compared to the HFHSD-fed 3xTg mice (17.09 ± 0.81 g) (Figure 1A, lower). Conversely, there was no statistically significant difference in body weight gain due to dietary curcumin in the NCD-fed 3xTg-AD mice (Figure 1A, upper; NCD:3.34 ± 1.06 g; NCD + CUR: 1.95 ± 1.07 g) or in both the NCD (NCD: 1.30 ± 0.90 g; NCD + CUR: 0.77 ± 2.01 g) and HFHSD-fed control mice (Figure 1A) at the end of intervention period. It is noteworthy that the HFHSD + CUR-fed control mice demonstrated a gradual weight gain, reaching 9.43 ± 0.92 g at week 13, contrasting with the rapid weight gain experienced by the HFHSD-fed control mice, which reached saturation within week 3 (11.44 ± 1.11 g) of HFHSD feeding (Figure 1A, lower). Importantly, the average body weight gain in the HFHSD-fed 3xTg-AD mice was 6.44 g higher than that of the control group, highlighting the susceptibility of the 3xTg-AD mice to obesity and metabolic diseases.

The study further explored the anticipated alterations in the insulin and glucose tolerance of the HFHSD + CUR-fed mice. However, curcumin supplementation did not lead to significant changes in the insulin and glucose tolerance (Figure 1B,C). In contrast, a reduced glucose tolerance was evident in the HFHSD-fed cohort of the 3xTg-AD group (blood glucose levels at 0, 15, and 120 min and AUC, *p* < 0.05) when compared to the HFHSD-fed control mice (Figure 1C, lower), indicating a heightened vulnerability of the 3xTg-AD mice to metabolic diseases. These findings suggest that while curcumin may mitigate weight gain, its effects on glucose metabolism in the context of HFHSD-induced metabolic challenges are complex and merit further investigation.

### 3.2. Curcumin Modulated the Hepatic Metabolism-Related Gene Expression in 3xTg-AD Mice

**Figure 2:**
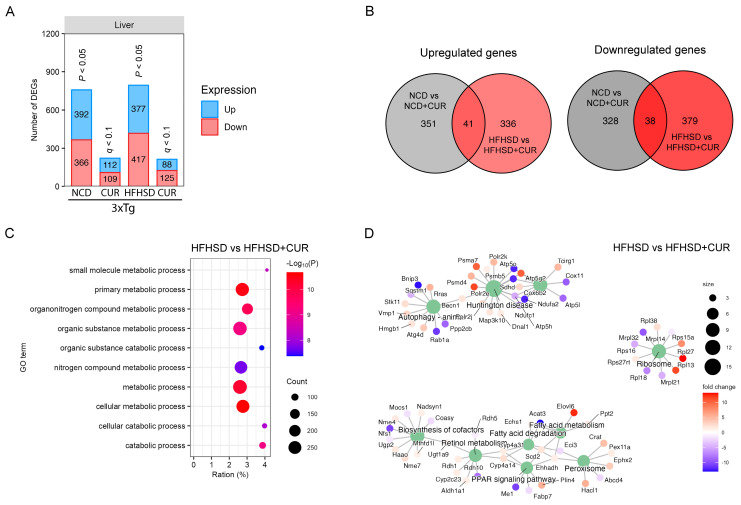
Hepatic transcriptome profiling using RNA sequencing for the 3xTg-AD mice fed with NCD and HFHSD. (**A**) Number of differentially regulated genes (DEGs) in the NCD and HFHSD diet-fed mice supplemented with and without curcumin (*n* = 4 per group for the NCD and NCD + CUR groups; *n* = 5 per group for the HFHSD and HFHSD + CUR groups). (**B**) Venn diagrams representing the overlapping and distinct DEGs between the different groups of the 3xTg-AD mice. (**C**) GO. (**D**) KEGG analysis for identifying the pathways altered by DEGs in the HFHSD-HFHSD + CUR-fed mice.

Given the liver’s pivotal role in metabolism, we conducted a comprehensive transcriptomic analysis of the liver tissues from the 3xTg-AD mice using RNA sequencing to elucidate the factors contributing to differential body weight gain. Curcumin supplementation led to a reduction in the number of differentially expressed genes (DEGs) in both the NCD-fed and HFHSD-fed 3xTg-AD mice (Figure 2A). Notably, the hepatic gene expression alterations induced by curcumin in the HFHSD-fed 3xTg-AD mice were distinct from those in the NCD-fed group, with 79 shared DEGs and 1394 unique DEGs between the two diet groups (Figure 2B).

To unravel the biological significance of the reduced DEGs under curcumin treatment, we performed a gene ontology (GO) term analysis, revealing a significant enrichment of the metabolism-related pathways in the HFHSD + CUR group, including the primary metabolic process, metabolic process, and cellular metabolic process (Figure 2C). Additionally, a KEGG pathway analysis unveiled substantial changes in peroxisome proliferator-activated receptor (PPAR) signaling, fatty acid metabolism, the biosynthesis of cofactors, ribosomes, Huntington’s disease, and autophagy pathway-related genes in the curcumin-supplemented HFHSD group (Figure 2D).

**Figure 3:**
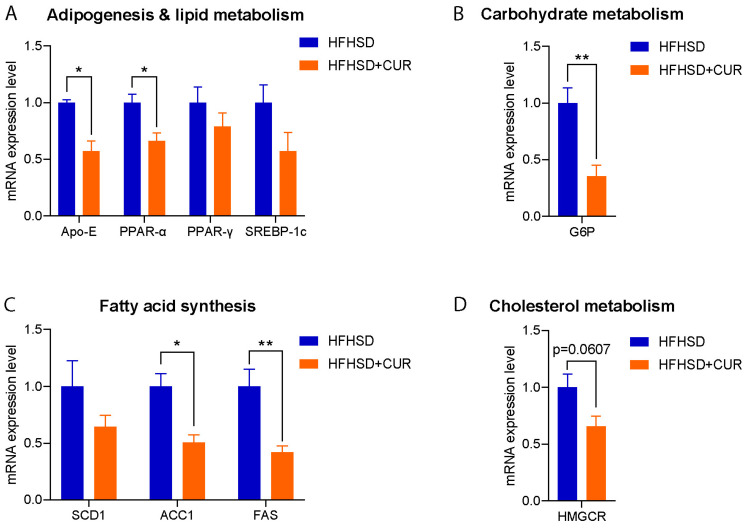
Relative expression of (**A**) adipogenesis and lipid metabolism, (**B**) carbohydrate metabolism, (**C**) fatty acid synthesis, and (**D**) the cholesterol metabolism-related genes in the liver of the HFHSD and HFHSD + CUR-fed 3xTg-AD mice (*n* = 3 per group). Curcumin reduced Acc1, PPARα, APOE, G6P, and FAS significantly in the HFHSD + CUR-fed 3xTg-AD mice. The results are expressed as the mean ± SEM and a significance difference was checked using an unpaired *t*-test at * *p* < 0.05, ** *p* < 0.01 vs. HFHSD-fed 3xTg mice.

To validate the observed reduction in body weight and understand the molecular basis, we assessed the relative expression of the genes related to lipid metabolism, adipogenesis, fatty acid synthesis, carbohydrate metabolism, and cholesterol metabolism in the liver of the HFHSD and HFHSD + CUR-fed 3xTg-AD mice. Remarkably, the expression of several genes associated with fatty acid synthesis and adipogenesis were significantly reduced in the curcumin-supplemented group compared to the HFHSD-fed 3xTg-AD group, validating the RNA sequencing results (Figure 3). Noteworthy genes such as Acc1, PPARα, APOE, G6P, and FAS exhibited a significant reduction, and SREBP-1c, SCD1, and PPARγ also showed a reduced expression upon curcumin supplementation. These findings provide robust support for the observed changes in body weight and transcriptional alterations in the pathways identified in the KEGG analysis, specifically in fatty acid metabolism and PPAR signaling in the curcumin-fed 3xTg-AD mice (Figure 2D).

### 3.3. Curcumin Attenuated HFHSD-Induced Impaired Spatial Recognition Memory in 3xTg-AD Mice

**Figure 4:**
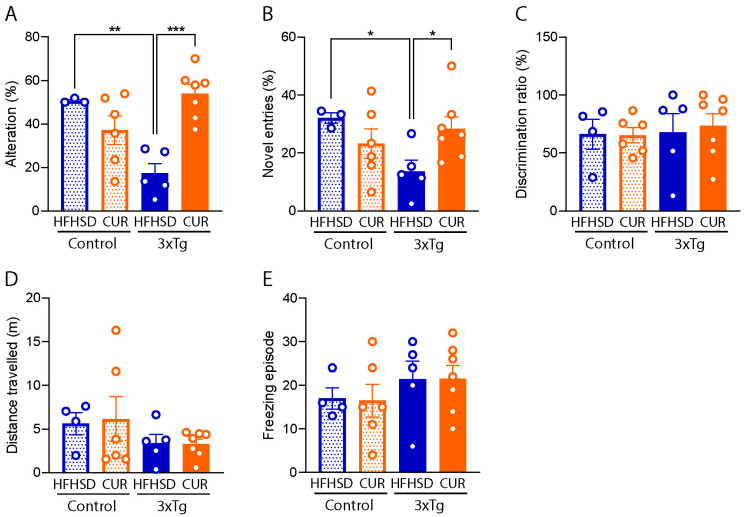
Behavioral evaluation of the HFHSD-fed group of the control and 3xTg-AD mice using the Y-maze test, NOR test, and OFT. (**A**) Spatial alteration in memory, (**B**) novel arm entries, (**C**) the discrimination ratio, (**D**) the distance traveled, and (**E**) the freezing episode observed on the HFHSD cohort of the 3xTg and control mice (*n* = 3 to 7 per group). The data are represented as the mean ± SEM and a significant difference in the percentage alteration between the group was checked using an ordinary one-way ANOVA following Tukey’s multiple comparison test at * *p* < 0.05, ** *p* < 0.01, *** *p* < 0.001. The novel entries between the groups were compared using an unpaired *t*-test at * *p* < 0.05. The mice that did not show positive weight gain from HFHSD feeding were excluded from the study.

To evaluate the influence of curcumin on memory function, we conducted comprehensive memory and locomotory function tests in the mice, employing the Y-maze, NOR test, and OFT. Our observations revealed a substantial decline in spatial memory function in the HFHSD-fed 3xTg-AD mice, as evidenced by both a reduced percentage alteration (Figure 4A; 17.3 ± 4.1%, *p* < 0.01) and fewer entries into the novel arms in the Y-maze test (Figure 4B; 13.7 ± 3.5%, *p* < 0.05), in comparison to the HFHSD-fed control mice. The percentage alteration was markedly lower (17.3 ± 4.1%) in the HFHSD-fed 3xTg mice, while curcumin supplementation in the 3xTg-AD group resulted in a substantial improvement (53.9 ± 3.9% *p* < 0.001), comparable to the HFHSD-fed control mice (50.6 ± 0.5%). The novel arm entries were also increased in the curcumin-supplemented HFHSD group (28.4 ± 3.9%, *p* < 0.05), contrasting with the 13.7 ± 3.5% recorded in the HFHSD-fed 3xTg group.

However, it is pertinent to note that no discernible behavioral differences were observed in the tested mice during the NOR test (Figure 4C) and OFT (Figure 4D,E). These findings suggest that curcumin supplementation specifically exerts a positive impact on spatial memory function, as indicated by the Y-maze results, without notable alterations in locomotory behavior or novel object recognition in the context of HFHSD-induced metabolic challenges in the 3xTg-AD mice.

### 3.4. Curcumin Modulated HFHSD-Induced Alteration in the Synaptic Plasticity and Mitochondrial Function-Related Pathways in the Hippocampus of 3xTg-AD Mice

**Figure 5:**
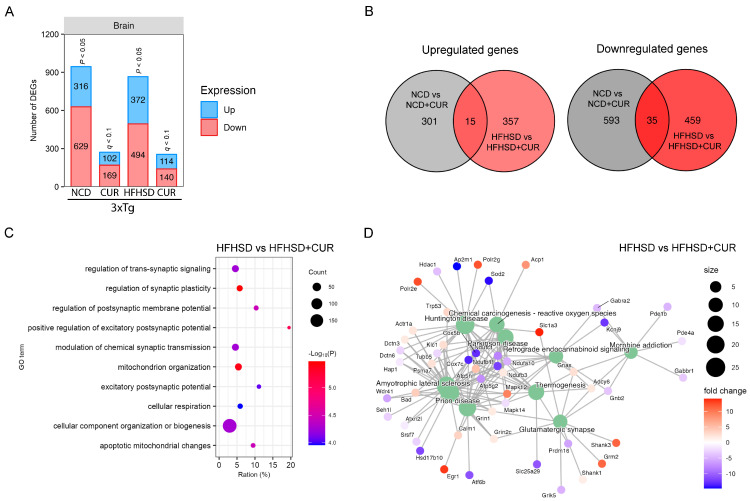
Brain transcriptome profiling using RNA sequencing for the 3xTg-AD mice fed with NCD and HFHSD. (**A**) Number of differentially regulated genes (DEGs) in the NCD and HFHSD diet-fed mice supplemented with and without curcumin (*n* = 3 to 4 per group). (**B**) Venn diagrams representing the overlapping and distinct DEGs between the different group of the 3xTg-AD mice. (**C**) GO analysis for identifying the pathways altered by DEGs in the HFHSD-HFHSD + CUR-fed group and (**D**) the KEGG pathway analysis for the HFHSD-HFHSD + CUR-fed mice.

Since the hippocampus plays a critical role in spatial memory formation, we conducted a comprehensive transcriptomic analysis of the hippocampal mRNA using RNA sequencing. Notably, curcumin induced a reduction in the number of DEGs in the hippocampus of both the NCD and HFHSD-fed mice (Figure 5A), mirroring the pattern observed in the liver (Figure 2A). In the comparison between the NCD and NCD + CUR groups, we identified a total of 945 DEGs, comprising 316 upregulated and 629 downregulated genes (Figure 5A). Similarly, in the comparison between the HFHSD and HFHSD + CUR groups, we observed 866 DEGs, with 372 upregulated and 494 downregulated genes (Figure 5A). The gene expression pattern due to curcumin supplementation between the NCD and HFHSD groups was notably distinct, with only 50 common DEGs and 1710 unique DEGs (Figure 5B).

To elucidate the functional implications of the reduced gene expression in the curcumin-treated groups, we conducted a GO analysis. Our results showed significant enrichment in the pathways related to the regulation of synaptic plasticity, the positive regulation of the excitatory postsynaptic potential, and mitochondrion organization, particularly in the HFHSD and HFHSD + CUR-fed mice (Figure 5C). Further exploration through the KEGG analysis revealed alterations in the genes related to glutamatergic synapses, thermogenesis, endocannabinoid signaling, Parkinson’s disease, Huntington’s disease, and prion disease-related genes in the HFHSD + CUR-fed mice (Figure 5D). These observations indicated that curcumin-induced changes in the hippocampal transcriptome may play a role in regulating the synaptic plasticity and excitatory postsynaptic potential, providing molecular insights into the observed improvements in spatial memory function, as evidenced by behavioral assessments.

### 3.5. Curcumin Improved the Microbiome Composition of the Gut in HFHSD-fed 3xTg-AD Mice

**Figure 6:**
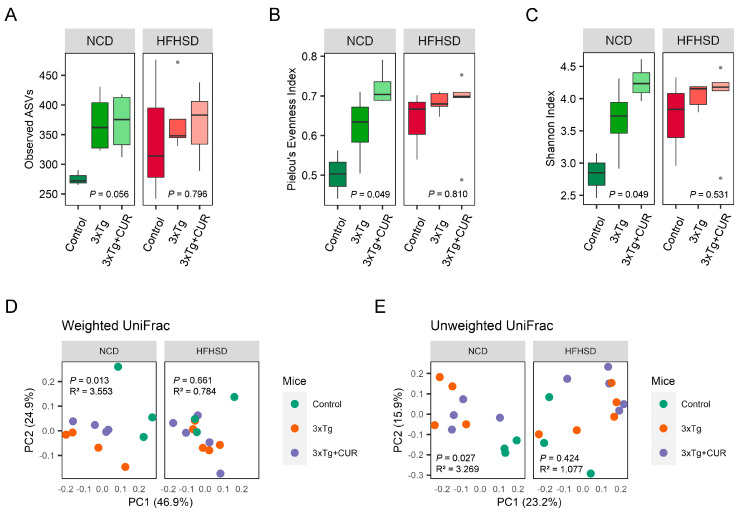
Effect of curcumin on the diversity of gut microbiota in the NCD and HFHSD-fed 3xTg-AD and control mice (*n* = 3 to 5). The alpha diversity is represented by the observed (**A**) ASVs, (**B**) Pielou’s evenness index, and the (**C**) Shannon index. The beta diversity was observed using a principal coordinate analysis (PCoA) based on the (**D**) weighted and (**E**) unweighted UniFrac distances.

In light of the existing evidence suggesting a potential link between the gut microbiome and AD, we sought to investigate the alterations in the gut microbiota following curcumin supplementation. Our observations revealed an overall increase in the alpha diversity of the microbiome in the 3xTg-AD mice compared to the control group This enrichment was reflected in the higher number of observed ASVs, an elevated Pielou’s evenness index, and an enhanced Shannon index (Figure 6A–C). To further assess the beta diversity of the microbiota community in the mouse feces, we conducted a principal coordinate analysis (PCoA) both quantitatively (using weighted UniFrac distances) and qualitatively (using unweighted UniFrac distances). The results indicated the influence of genetics, diet, and curcumin on the taxa level of the microbiome in the mouse feces (Figure 6D,E).

**Figure 7:**
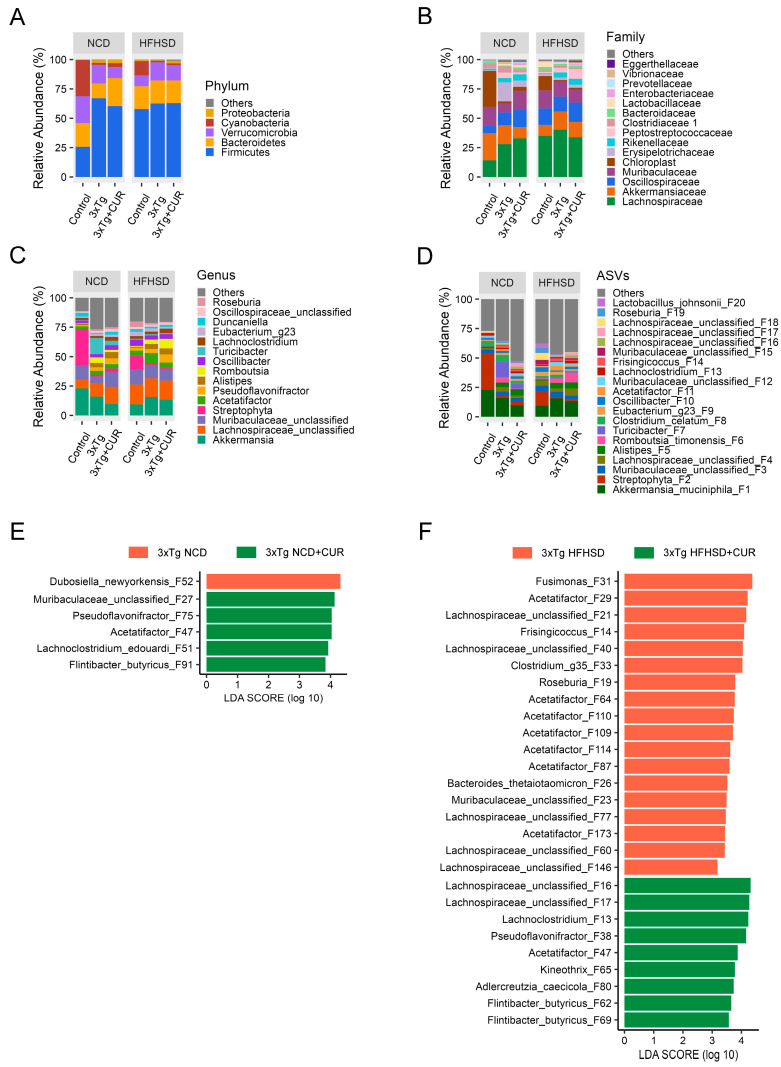
Effect of curcumin on the gut microbiome composition of the NCD and HFHSD-fed 3xTg-AD and control mice (*n* = 3 to 5). An analysis was performed in at the ASV level to find alterations in the (**A**) phyla, (**B**) family, and (**C**,**D**) genus levels. The differential enrichment of bacterial ASVs between (**E**) NCD and (**F**) HFHSD fed cohort of 3xTg mice was determined using the linear discriminant analysis (LDA) effect size (LEfSe), with the all-against-all multiclass analysis, *p* < 0.05, and a logarithmic LDA threshold of 3.0.

At the phylum level, we did not observe major changes in the most abundant microbiome, except for *Verrucomicrobia*. Notably, the abundance of *Verrucomicrobia* decreased in the curcumin-fed mice (Figure 7A). Moreover, we observed the restoration of some beneficial gut microbiota in the curcumin-supplemented mice at the family and genus levels, regardless of whether they were fed a NCD or a HFHSD. At the family level, two different bacteria were enriched, namely, *Oscillospiraceae*, and *Rikenellaceae* (Figure 7B), and at the genus level, an abundance of five different bacteria, *Oscillibacter*, *Alistipes, Pseudoflavonifractor*, *Duncaniella*, and *Flintibacter* (Figure 7C–F), were increased.

## 4. Discussion

In our investigation, curcumin supplementation demonstrated a notable impact on body weight gain in the 3xTg-AD mice subjected to HFHSD. This aligned with the previous findings from our studies and others, revealing a consistent reduction in body weight in the mice following curcumin supplementation, particularly under various metabolic challenges [29,30,31]. However, it is essential to note that the effects of cumulative weight gain in the HFHSD-fed control mice were evident only in the initial weeks of intervention, with no significant reduction observed at the study’s conclusion. The distinct body weight gain patterns observed among the groups and the nuanced effects of curcumin may be attributed to gender and genetic variations [2,3,35,36]. Regardless, curcumin consistently mitigated the rate of weight gain in both the control and 3xTg-AD groups under HFHSD conditions.

HFHSD consumption is commonly associated with impaired insulin sensitivity and insulin resistance, particularly exacerbating metabolic syndrome in vulnerable individuals [37]. While 3xTg-AD female mice are considered more susceptible to insulin-related metabolic syndrome [38], our ITT results did not reveal any significant differences between the 3xTg-AD and control mice, with no noticeable impact from curcumin supplementation (Figure 1B). These results may be attributed to genetic, age, and dietary differences [3,38]. The GTT further indicated a higher baseline glucose level in the HFHSD-fed 3xTg-AD mice, emphasizing their vulnerability to diabetes (Figure 1C). Unfortunately, curcumin did not significantly improve the increased glucose levels and glucose tolerance in the HFHSD-fed 3xTg-AD mice, suggesting that the observed reduction in body weight and memory improvement may not be directly associated with improved insulin signaling. This finding was in accord with our previous study, where we did not observe an effect of curcumin on the glucose tolerance in mice [31], despite findings from other researchers indicating an increased glucose tolerance in curcumin-treated mice [39,40,41]. This discrepancy might be attributed to differences in the dose, research design, age, and genetics of the mice.

Driven by the curiosity surrounding the selective impact of curcumin on the body weight in the HFHSD-fed 3xTg-AD mice, we performed a comprehensive transcriptomic analysis of the liver. Intriguingly, we observed a significant reduction in the gene expression after curcumin treatment, particularly evident in the altered metabolic pathways, including those related to fatty acid metabolism and PPAR signaling. The downregulation of the genes associated with adipogenesis, fat accumulation, and the obesity-related pathways was further validated by the qPCR results, emphasizing the beneficial impact of curcumin on reducing the cumulative body weight and improving the metabolic profiles in response to the downregulation of those genes [42,43,44,45,46,47,48,49].

The 3xTg-AD mice served as crucial models for studying AD pathology due to their overexpression of the APP, tau, and Psen1 genes associated with familial AD [50]. Female mice have even been found to be more susceptible to amyloid pathology, beta secretase activity [51], and behavioral decline compared to males, making them an ideal AD model [52]. Lifestyle factors, including diet, play pivotal roles in manifesting AD symptoms in these mice. The worsening of memory function under HFHSD was evident in our study, as demonstrated by a reduced performance during the Y-maze test (Figure 4). Curcumin supplementation effectively ameliorated these memory deficits, as evidenced by an enhanced performance during the Y-maze test, consistent with our prior finding [53]. However, no significant differences were observed in the OFT and NOR test, suggesting selective improvements in spatial memory. To explore the potential molecular mechanisms underlying the cognitive improvements from our observations, we conducted a transcriptomic analysis of the hippocampus. The results indicated a reduction in the number of DEGs after curcumin supplementation, particularly related to the pathways involved in the regulation of synaptic plasticity, the positive regulation of excitatory postsynaptic potential, and mitochondrial organization. These findings suggest that curcumin-induced changes in the hippocampal transcriptome may contribute to the observed improvements in spatial memory function.

Given the emerging role of the gut microbiome in influencing various diseases, including Alzheimer’s, we examined the changes in gut microbiota following curcumin supplementation [54]. The link between gut microbiota and various diseases, as well as the microbiome involvement in immunity, metabolism, inflammation, amyloid, and tau pathology, has made this area of research critical [24,54,55]. The evidence supporting the role of the gut microbiome in AD pathology includes studies where the colonization of 3xTg-AD mice with fecal samples from AD patients exacerbated AD outcomes compared to those colonized with feces from healthy donors [56]. Notable changes were found in the abundance of several gut microbiota at different taxonomic levels in our study. Curcumin supplementation led to a reduction in the abundance of *Verrucomicrobia* and an increase in beneficial bacteria at the family and genus levels, such as *Oscillospiraceae*, *Rikenellaceae*, *Oscillibacter*, *Alistipes*, *Pseudoflavonifractor*, *Duncaniella*, and *Flintibacter.* These microbiomes were associated with an improved metabolic syndrome, reduced inflammation, and potential positive roles in the liver and cardiovascular health, as shown in previous studies [57,58,59,60,61,62,63,64,65].

While our study provides valuable insights into the multifaceted effects of curcumin on body weight, metabolic pathways, memory function, and gut microbiota, it is essential to acknowledge some limitations. The limited number of mice in each group may have introduced individual variations, and future studies with larger cohorts could enhance the robustness of the findings. Additionally, the quantification of short-chain fatty acids and inflammatory cytokines in the serum could provide a more comprehensive understanding of the systemic changes induced by altered microbiota populations.

## 5. Conclusions

In conclusion, our study demonstrated the multifaceted effects of curcumin on the body weight, metabolic pathways, memory function, and gut microbiota in 3xTg-AD mice under HFHSD conditions. The selective reduction in body weight gain, improvements in memory function, and the modulation of the metabolic pathways suggest the potential therapeutic value of curcumin in mitigating Alzheimer’s-related symptoms. The observed changes in the gut microbiota further underscored the intricate interplay between the gut–brain and gut–liver axes. Future research may benefit from a large-scale study exploring the effects of curcumin in both animal models and human participants. Additionally, a clearer understanding could be gained from the impact of individual microbiome changes using germ-free mice, quantifying short-chain fatty acids, and assessing gut permeability. These approaches would contribute to a more precise interpretation of alterations in specific bacterial populations.

## Data Availability

The raw sequencing reads for both liver and RNA sequencing, and 16S rRNA sequencing were deposited in the NCBI Sequence Read Archive database under the same BioProject accession number PRJNA1052364. All the datasets used and/or analyzed during the current study are available from the corresponding authors upon reasonable request. The data are not publicly available due to privacy.
